# Hunger and Behavioral Risk Factors for Noncommunicable Diseases in School-Going Adolescents in Bolivia, 2012

**DOI:** 10.5888/pcd13.160015

**Published:** 2016-04-21

**Authors:** Matthew L. Romo

**Affiliations:** Author Affiliations: Dr Romo is also affiliated with the Dirección de Investigación, Universidad de Cuenca, Cuenca, Ecuador.

## Abstract

Hunger may play a role in noncommunicable disease (NCD) risk. This study used the 2012 Global School-based Student Health Survey from Bolivia to determine the association between hunger and risk factors for NCDs among adolescents. Hunger was associated with increased odds of nondaily fruit and vegetable consumption (adjusted odds ratio [AOR] = 1.21; *P* < .001), inadequate physical activity (AOR = 1.21; *P* = .001), and current tobacco use (hunger sometimes [AOR = 1.83; *P* < .001] or most of the time/always [AOR = 2.12; *P* < .001]). Interventions to reduce the burden of NCDs in Bolivia should address hunger, in addition to traditional behavioral risk factors.

## Objective

Bolivia has the highest prevalence of chronic undernourishment in South America, and it affects 15.9% of the Bolivian population (1). Hunger is an individual-level condition that may result from household food insecurity (2). Hunger and household food insecurity have been associated with noncommunicable diseases (NCDs) and may cluster with other risk factors (3–5). Although the communicable disease burden is substantial in Bolivia, NCDs account for the greatest mortality; an estimated 59% of total deaths are attributable to NCDs in Bolivia (6). Understanding the associations between NCDs and hunger may provide insight into ways to reduce risk for NCDs, particularly among adolescents, because of the potential for early intervention before NCD onset. The objective of this study was to determine the association between hunger and behavioral risk factors for NCDs among school-going adolescents in Bolivia.

## Methods

The 2012 Global School-Based Student Health Survey (GSHS) for Bolivia was a nationally representative survey of school-going adolescents, using a clustered sample design that first sampled schools and then classes (7). Participation in the survey was voluntary. The survey was self-administered in Spanish and was anonymous, with ethical oversight maintained by the Ministry of Health in Bolivia.

Hunger was defined as going hungry in the past 30 days because of not enough food in the home. Possible answers were “never,” “rarely,” “sometimes,” “most of the time,” or “always,” with the last 2 answers combined because of small numbers in each category. Demographic variables were self-reported age and sex, and region of residence. Students were asked how many vegetables, fruits, and sugar-sweetened sodas they consumed daily in the previous 30 days; answers were categorized as daily consumption or nondaily consumption. Current tobacco use and alcohol use were defined as having used either substance on at least one day in the past 30 days. Inadequate physical activity was defined as less than 5 days of 60 minutes or more per day in the previous 7 days (8). Obesity was defined as having a body mass index more than double the standard deviation of the median for age and sex from the 2007 World Health Organization child growth reference data (9).

Rao-Scott χ^2^ tests and multivariable logistic regression were used to assess the significance of associations of variables of interest with hunger. Multiple imputation was used to determine bias of missing data in the multivariable models (20 imputations, fully conditional specification).

The significance level was set at a 2-sided α of .05. All analyses were performed in SAS 9.4 (SAS Institute Inc.) and adjusted for the selection probabilities related to the complex sample design and weighted to the sex and grade distribution of the school-going adolescent population of Bolivia.

## Results

The overall survey response rate (school response rate multiplied by student response rate) was 88%, with 3,696 students participating. Experiencing any hunger in the previous 30 days was reported by 61.9% of adolescents (Table 1) and was most prevalent among students aged 16 years or older (64.2%), boys (64.0%), and residents of the valley region (66.8%). There were significant differences by hunger status in the distribution of fruit and vegetable consumption (*P* < .001), current tobacco use (*P* < .001), current alcohol use (*P* = .03), and physical activity (*P* = .001) but not in sugar-sweetened soda consumption (*P* = .56) or obesity (*P* = .53).

**Table 1:** Bivariate Analysis of Association Between Hunger Frequency and Behavioral Risk Factors for Noncommunicable Diseases Among School-Going Adolescents (N = 3,696), Global School-Based Student Health Survey, Bolivia, 2012

Characteristic	Category Total, n (%^a^)	Hunger Frequency				
		Never, n (%^a^)	Rarely, n (%^a^)	Sometimes, n (%^a^)	Most of the Time/Always, n (%^a^)	*P *Value^c^
**All respondents, n (%)**	3,647^b^ (100)	1,358 (38.1)	1,264 (34.5)	695 (18.7)	330 (8.6)	—
**Age, y (n = 3,501^b^)**						
≤13	860 (23.1)	352 (41.3)	270 (31.3)	139 (16.1)	99 (11.4)	<.001
14	989 (27.3)	365 (38.5)	361 (36.0)	171 (16.6)	92 (8.8)	
15	966 (29.0)	361 (37.8)	346 (36.1)	201 (20.5)	58 (5.5)	
≥16	686 (20.6)	234 (35.8)	240 (34.4)	150 (21.1)	62 (8.8)	
**Sex (n = 3,496^b^)**						
Female	1,719 (48.8)	683 (40.8)	577 (32.8)	335 (19.5)	124 (6.9)	.008
Male	1,777 (51.2)	620 (36.0)	642 (36.4)	330 (17.8)	185 (9.8)	
**Region (n = 3,647^b^)**						
Highlands (La Paz, Oruro, Potosí)	1,411 (45.2)	512 (35.6)	506 (36.9)	245 (17.3)	148 (10.2)	<.001
Plains (Santa Cruz, Beni, Pando)	1,013 (33.6)	444 (44.7)	305 (29.7)	195 (19.7)	69 (5.9)	
Valley (Cochabamba, Chuquisaca, Tarija)	1,223 (21.2)	402 (33.2)	453 (37.0)	255 (20.2)	113 (9.5)	
**Fruit and vegetable consumption (n = 3,608^b^)**						
Nondaily	1,428 (39.4)	438 (31.6)	522 (36.3)	335 (23.3)	133 (8.8)	<.001
Daily	2,180 (60.6)	907 (42.5)	733 (33.6)	351 (15.7)	189 (8.2)	
**Sugar-sweetened soda consumption (n = 3,635^b^)**						
Daily	2,273 (63.0)	870 (39.2)	770 (33.7)	433 (18.7)	200 (8.4)	.56
Nondaily	1,362 (37.0)	485 (36.5)	489 (35.8)	261 (18.8)	127 (8.9)	
**Current tobacco use (n = 3,581^b^)**						
Yes	592 (17.2)	173 (29.2)	194 (33.5)	150 (25.4)	75 (11.9)	<.001
No	2,989 (82.8)	1,167 (40.3)	1,048 (34.8)	530 (17.2)	244 (7.7)	
**Current alcohol use (n = 3,514^b^)**						
Yes	642 (18.9)	207 (32.3)	241 (37.8)	142 (21.7)	52 (8.1)	.03
No	2,872 (81.1)	1,110 (39.8)	973 (33.6)	528 (18.0)	261 (8.6)	
**Physical activity (n = 3,604^b^)**						
Inadequate	2,794 (76.6)	998 (36.4)	978 (34.8)	563 (19.9)	255 (8.8)	.001
Adequate	810 (23.4)	345 (43.6)	272 (33.9)	123 (14.8)	70 (7.7)	
**Obesity (n = 3,324^b^)**						
Yes	161 (4.7)	66 (40.4)	51 (29.9)	31 (22.1)	13 (7.7)	.53
No	3,163 (95.3)	1,181 (38.5)	1,114 (35.1)	594 (18.3)	274 (8.2)	

^a^ All percentages were weighted according to the sex and grade distribution of adolescent students in Bolivia. Row percentages may not total 100% because of rounding.

^b^ Number of participants is reduced because of list-wise deletion (ie, any participants with missing data were excluded).

^c^ Rao-Scott χ^2 ^test used to calculate *P* values.

In multivariable logistic regression models adjusted for age, sex, region, and all other behavioral risk factors, any hunger versus no hunger was significantly associated with greater odds of nondaily fruit and vegetable consumption (adjusted odds ratio [AOR] = 1.21; 95% confidence interval [CI], 1.11–1.31; *P* < .001), inadequate physical activity (AOR = 1.21; 95% CI, 1.08–1.35; *P* = .001), and current tobacco use (AOR = 1.32; 95% CI, 1.18–1.47; *P* < .001), but not daily sugar-sweetened soda consumption (AOR = 0.95; 95% CI, 0.86–1.04; *P* = .23), current alcohol use (AOR = 0.95; 95% CI, 0.84–1.07; *P* = .38), or obesity (AOR = 0.97; 95% CI, 0.77–1.21; *P* = .76). When evaluating frequency of hunger (Table 2), an increasing exposure–response effect was not observed for nondaily fruit and vegetable consumption or adequate physical activity, but there was a possible exposure–response effect for current tobacco use (experiencing hunger rarely [AOR = 1.21; 95% CI, 0.93–1.56; *P* = .15], sometimes [AOR 1.83; 95% CI: 1.34–2.50; *P* = <.001], or most of the time/always [AOR = 2.12; 95% CI, 1.50–2.99; *P* < .001]). Multiple imputation confirmed the overall magnitude and precision of these results.

**Table 2:** Multivariable Logistic Regression Analysis^a^, Association Between Hunger Frequency and Behavioral Risk Factors for Noncommunicable Diseases Among School-Going Adolescents (N = 3,696), Global School-Based Student Health Survey, Bolivia, 2012

Characteristic	Nondaily Fruit and Vegetable Consumption AOR (95% CI) [*P* Value]	Daily Sugar-Sweetened Soda Consumption AOR (95% CI) [*P* Value]	Current Tobacco Use AOR (95% CI) [*P* Value]	Current Alcohol Use AOR (95% CI) [*P* Value]	Inadequate Physical Activity AOR (95% CI) [*P* Value]	Obesity AOR (95% CI) [*P* Value]
**Complete Case Analysis**						
**Hunger frequency (n = 3,094^b^)**						
Never	1.00 [Reference]	1.00 [Reference]	1.00 [Reference]	1.00 [Reference]	1.00 [Reference]	1.00 [Reference]
Rarely	1.41 (1.22–1.63) [<.001]	0.84 (0.67–1.06) [.13]	1.21 (0.93–1.56) [.15]	1.21 (0.94–1.57) [.14]	1.33 (1.09–1.61) [.005]	0.88 (0.57–1.36) [.58]
Sometimes	1.85 (1.44–2.38) [<.001]	0.92 (0.72–1.17) [.49]	1.83 (1.34–2.50) [<.001]	1.04 (0.70–1.54) [.85]	1.64 (1.18–2.29) [.004]	1.00 (0.53–1.86) [.99]
Most of the time/always	1.32 (1.01–1.73) [.04]	0.82 (0.62–1.09) [.18]	2.12 (1.50–2.99) [<.001]	0.69 (0.41–1.14) [.15]	1.48 (1.03–2.13) [.04]	0.84 (0.40–1.75); [.64]
**Multiple Imputation Analysis**						
**Hunger frequency (n = 3,696)**						
Never	1.00 [Reference]	1.00 [Reference]	1.00 [Reference]	1.00 [Reference]	1.00 [Reference]	1.00 [Reference]
Rarely	1.39 (1.20–1.62) [<.001]	0.89 (0.72–1.10) [.28]	1.08 (0.83–1.41) [.55]	1.25 (0.97–1.62) [.09]	1.27 (1.04–1.57) [.02]	0.81 (0.52–1.24) [.31]
Sometimes	1.85 (1.50–2.30) [<.001]	0.95 (0.74–1.22) [.68]	1.97 (1.48–2.63) [<.001]	1.01 (0.71–1.44) [.95]	1.63 (1.19–2.24) [.003]	1.11 (0.64–1.93) [.70]
Most of the time/always	1.41 (1.08–1.83) [.01]	0.89 (0.68–1.16) [.37]	2.30 (1.64–3.23) [<.001]	0.79 (0.50–1.25) [.31]	1.45 (1.02–2.06) [.04]	0.82 (0.42–1.60) [.56]

Abbreviations: AOR, adjusted odds ratio; CI, confidence interval.

^a^ Each of the 6 models was adjusted for age, sex, region, and all dependent variables of interest (nondaily fruit and vegetable consumption, daily sugar-sweetened soda consumption, current tobacco use, current alcohol use, inadequate physical activity, and obesity), excluding the independent variable modeled. ^b^ Number of participants is reduced because of list-wise deletion (ie, any participants with missing data were excluded).

## Discussion

Hunger from lack of food in the home was highly prevalent and was significantly associated with various risk factors for NCDs, potentially representing pathways in which hunger contributes to the burden of NCDs. Geographic differences were also relevant, because the valley and highland regions (highest hunger prevalence) have the most people living in poverty, and the plains region (lowest hunger prevalence) has the fewest people living in poverty (10).

The association between hunger and nondaily fruit and vegetable consumption and inadequate physical activity is supported by research finding associations with household food insecurity (4,11). Although these associations were independent, these variables might be at least partially related, because insufficient access to high-nutrient–dense foods in general could lead to nutrient deficiencies and poorer general health, which might impede participation in physical activity.

Adolescents reporting hunger sometimes or most of the time or always had approximately 2 times the odds of being current tobacco users. This association is supported by a study among US Latino adults, which found that current smokers had significantly greater odds of household food insecurity than did never smokers (5). Many explanations are possible as to why adolescents who reported hunger used tobacco; for example, using tobacco may be a coping mechanism for psychological stress resulting from hunger and poverty.

Limitations of this study are its cross-sectional design, self-reporting of data, and lack of data on adolescents who do not attend school or were not present on the day of the survey. School attendance is particularly relevant in Bolivia where a substantial proportion of children are in the workforce.

This study highlights several opportunities for public health interventions in Bolivia. Because hunger was associated with nondaily fruit and vegetable consumption and inadequate physical activity, school meal programs and physical education need to be scaled up, particularly in the poor, rural parts of the country, and the feasibility of weekend school meal programs also needs to be explored. At the same time, policy-level interventions are needed, especially for tobacco pricing. Cigarettes in Bolivia remain remarkably inexpensive (12) and highly accessible to all, including those living in poverty.
